# Temperature Sensing in Agarose/Silk Fibroin Translucent Hydrogels: Preparation of an Environment for Long-Term Observation

**DOI:** 10.3390/nano15020123

**Published:** 2025-01-16

**Authors:** Maria Micheva, Stanislav Baluschev, Katharina Landfester

**Affiliations:** 1 Max Planck Institute for Polymer Research, Ackermannweg 10, 55128 Mainz, Germany; michevam@mpip-mainz.mpg.de (M.M.); landfester@mpip-mainz.mpg.de (K.L.); 2 Faculty of Physics, Sofia University “St. Kliment Ohridski”, 5 James Bourchier Blvd., 1164 Sofa, Bulgaria

**Keywords:** annihilation upconversion, minimally invasive, agarose/silk fibroin hydrogel, oxygen sensing, temperature sensing, cell culture

## Abstract

Environmental changes, such as applied medication, nutrient depletion, and accumulation of metabolic residues, affect cell culture activity. The combination of these factors reflects on the local temperature distribution and local oxygen concentration towards the cell culture scaffold. However, determining the temporal variation of local temperature, independent of local oxygen concentration changes in biological specimens, remains a significant technological challenge. The process of triplet–triplet annihilation upconversion (TTA-UC), performed in a nanoconfined environment with a continuous aqueous phase, appears to be a possible solution to these severe sensing problems. This process generates two optical signals (delayed emitter fluorescence (dF) and residual sensitizer phosphorescence (rPh)) in response to a single external stimulus (local temperature), allowing the application of the ratiometric-type sensing procedure. The ability to incorporate large amounts of sacrificial singlet oxygen scavenging materials, without altering the temperature sensitivity, allows long-term protection against photo-oxidative damage to the sensing moieties. Translucent agarose/silk fibroin hydrogels embedding non-ionic micellar systems containing energetically optimized annihilation couples simultaneously fulfill two critical functions: first, to serve as mechanical support (for further application as a cell culture scaffold); second, to allow tuning of the material response window to achieve a maximum temperature sensitivity better than 0.5 K for the physiologically important region around 36 °C.

## 1. Introduction

Intracellular oxygen concentration, local acidity, and local temperature in living objects are regulated and maintained within narrow physiological limits. Any change in these factors indicates a deviation from typical cellular metabolism, making them valuable factors to monitor [1]. A variety of tools are available to sense temperature, such as fluorescent nanodiamonds [2], a variety of thermometers at the micro-nanoscales [3], electrochemical sensing [4], and temperature-responsive fluorescent materials [5]. Oxygen sensing is also essential for the survival of biological objects, either to understand many physiological processes or to diagnose diseases [6]. Several techniques such as single nanoparticles with a ratiometric O_2_-response [7] or phosphorescent metalloporphyrins encapsulated in hydrophobic dendrimers [8] can be used for this purpose. However, the major experimental drawback of these sensing techniques is that phosphorescence emission is an integral parameter that simultaneously depends on the local temperature and local oxygen contamination [9]. Oxygen concentration can be sensed by single intensity-based methods [10,11,12]. More reproducible sensing measurements have been obtained by applying ratiometric techniques, where the oxygen concentration dependence of the fluorescence of blue emitting polyfluorene is compared with the dependence of the phosphorescence signal of platinum porphyrin [7,13]. Both reported experiments use a blue emitting diode laser, λ = 405 nm, as the excitation source, whose interaction with the living object cannot be neglected.

An alternative process that can be used for sensing the two parameters—temperature and oxygen—is TTA–UC. The process of TTA-UC has a number of different applications such as renewable energy sources [14,15,16,17,18], molecular sensing [19], and biological imaging [20]. Another important reason to use the TTA-UC is the low excitation intensities (at the level of 1 W·cm^−2^, red excitation light, λ > 630 nm) that are required for this process, so the radiation stress for the biological samples is kept to a low level. 

**Figure 1:**
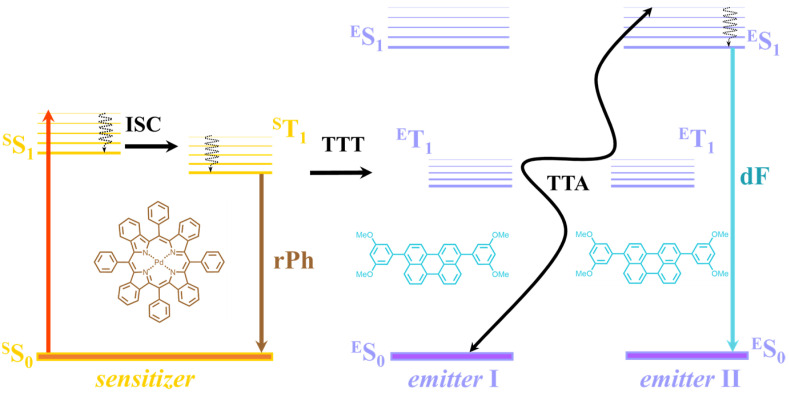
Simplified energetic scheme of the triplet–triplet annihilation upconversion (TTA–UC) process in an oxygen-free environment. Insets: structure of meso-tetraphenyl-tetrabenzo porphyrin palladium(II) (PdTBP) sensitizer (**left**), structure of 3,10-bis(3,5-dimethoxyphenyl)perylene (BDMP) emitter (**right**).

TTA–UC is a process that takes place in a multi-chromophore system consisting of energetically optimized pairs of sensitizer and emitter molecules. Photon energy is absorbed by a sensitizer and stored in its triplet state formed in the process of intersystem crossing (ISC). Afterwards, this energy is transformed into an emitter triplet state through the process of triplet–triplet transfer. Next, the excited triplet states of two emitter molecules undergo triplet–triplet annihilation (TTA), in which one emitter molecule returns back to its singlet ground state and the other molecule gains the energy of both triplet states and is excited to the higher singlet state. As the singlet state emitter radiatively decays back to the ground state, a dF (the blue arrow, Figure 1), bearing higher energy than that of the excitation photon, is emitted. At the same time, if the energy overlap between the triplet manifolds of the emitter and sensitizer molecules is not optimal, the triplet state of the sensitizer will not be completely depopulated and, therefore, rPh (the brown arrow, Figure 1) will be observed [21,22,23,24].

The process of TTA–UC is based on optically created densely populated organic triplet ensembles, in which the intermolecular energy transfer strongly depends on the local temperature, the local viscosity, and the amount of molecular oxygen present. The processes based on energy transfer between excited triplet states are chosen because of their long lifetimes and the pronounced interaction of these triplet ensembles with the environment. The dependence of the TTA–UC process on the sample temperature can be related to the increased molecular mobility [25]. Furthermore, if the molecular mobility of the interacting organic species is not optimal, the sensitizer triplet state will not be completely depopulated and, therefore, the dF and sensitizer’s rPh signals will be simultaneously observed. Consequently, the increase in molecular mobility leads to a significant increase in the intensity of the emitter’s delayed fluorescence and a simultaneous decrease in the residual sensitizer’s phosphorescence.

All ongoing processes in the annihilation upconversion system are associated with the triplet states of the sensitizer and the emitter molecules. The lifetime of these processes measures up to tens or even hundreds of microseconds [26], which is more than enough for the excited triplet states of the sensitizer and the emitter molecules to be affected by the presence of molecular oxygen. The interaction of the excited triplet states of the participating molecules and the oxygen in the triplet ground state results in the effective generation of singlet oxygen [27].

Therefore, in life science objects, where oxygen is always present, the quenching rates of the optical signals associated with the TTA–UC process are uncertain. For this reason, a proposed solution to the quenching problem is the addition of a sacrificial singlet oxygen scavenger (SSOS) material to the TTA-UC composition. However, there are ultimate requirements that a particular molecule/material has to fulfill to be considered an SSOS. (i) The SSOS molecules must chemically bind only the singlet oxygen present in the sensing structure. This requirement is of critical importance, e.g., if the SSOS material acts as a common antioxidant (i.e., it chemically binds even the molecular oxygen in the ground state), firstly, it will prevent breathing of the living species and, secondly, the O_2_-sensing function will be lost [28]. Furthermore, the formed oxidation products must be chemically stable for the temperature range of interest (T_max_ < 50 °C, if the T-sensing is applied to biological samples [29]). (ii) The SSOS molecules must show pronounced hydrophobicity and be well miscible with organic solvents. This requirement is fundamental. First, the TTA–UC process has been demonstrated up to now only in a hydrophobic microenvironment and, second, the SSOS molecules must not interfere with the cell metabolism (water-soluble SSOS materials will penetrate into the cell culture environment, causing unpredictable changes in its properties). (iii) The SSOS molecules must demonstrate relatively low viscosity and ideally be in a liquid state for the temperature range of interest. (iv) The SSOS molecules must be inherently biocompatible and non-toxic.

In order to achieve TTA–UC in a water macro-environment (continuous phase), it is necessary to embed the active moieties in a hydrophobic micro-environment (dispersed phase). Previously, embedding of the UC was demonstrated (materials in oil-in-water microemulsions [30], polymer nanocapsules [24] or oil-laden microcapsules [31], multiphasic protein hydrogels [32], or enzymatic environments [33]).

To fully explore the sensing properties of the TTA–UC process, the composition of the optically active materials must be optimized for two different, somewhat contradictory, requirements. T-sensitivity depends on the steep increase in the molecular mobility of the UC-active molecules as the local temperature increases. In addition, the requirement to demonstrate the highest possible T-sensitivity for a given temperature range, centered around the physiologically important value of 36 °C, defines a certain mixture of hydrophobic materials with high melting points (beeswax, 62 °C; carnauba wax, 82 °C) or low melting points (natural oils, characterized by low viscosity). On the other hand, the long-term protection against photo-oxidation requires embedding of the nanoconfined sensors’ large amount of SSOS materials (often oils, bearing high amounts of unsaturated double bonds [27]). Due to these partially contradictory requirements, the demonstrated T-sensitivity in organogel matrices seems to be less than optimal, while much higher T-sensitivity can be achieved in micellar systems. 

Therefore, in order to optimize nanoconfined systems in an independent manner, two types of micelles, one dedicated to the T-response and the other embedding the SSOS materials and responsible for the singlet oxygen protection, are used. 

However, the micellar structures have low mechanical stability, where any physical contact with soft matter surfaces (including cell membranes) leads to the inevitable destruction of the micelle. To ensure mechanical stability, the micelles are embedded in agarose/silk fibroin hydrogels. The gel support has to fulfill a chain of requirements. (i) First of all, the gel must be highly hydrated in order to prevent changes in the photophysical properties; (ii) the gel layer itself must remain approximately unchanged with the increase in temperature, which defines the use of two ingredients with opposite responses to the temperature increase. Even if the working temperature range is much lower than the gelation temperature of the specific type of agarose [34] used, the agarose gel swells. In contrast, silk fibroin gels [35] wrinkle faster with the temperature increase. The combination of both materials allows the synthesis of a supporting hydrogel with sustainable physical parameters and optical properties over the temperature window of interest. 

Briefly, we follow the experimental route, including (i) the preparation of a translucent agarose/silk fibroin hydrogel embedding non-ionic micellar systems containing energetically optimized annihilation couples; (ii) enrichment of the nanoconfined sensing moieties with large amounts of SSOS materials, allowing long-term and reproducible operation of the TTA-UC molecular system (even in oxygen-saturated environments); (iii) effective binding of the locally generated singlet oxygen allowing to achieve relative stationary intensities of the optical signals of dF and rPh; (iv) increasing the sample temperature, causing a significant increase in the intensity of the delayed fluorescence of the emitter and a concomitant decrease in the residual phosphorescence of the sensitizer, allowing a ratiometric temperature calibration curve to be established; (v) two types of micelles, one dedicated to the T-response and the other embedding the SSOS materials and responsible for the singlet oxygen protection, allow an independent optimization of the nanoconfined sensing system, demonstrating a high sensitivity for the physiologically relevant temperature range ∆T_physio_ = 30–42 °C. 

## 2. Materials and Methods

### 2.1. Materials

**Figure 2:**
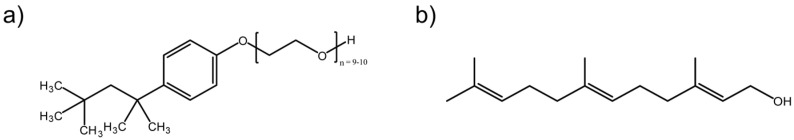
Chemical structures of (**a**) Triton X-100 and (**b**) farnesol.

Surfactant Triton X-100 (HLB = 13.4), farnesol, DMEM (Dulbecco’s modified Eagle’s medium), agarose (medium electroendosmosis, EEO, M_w_ = 630.55 g/mol) and silk fibroin, and 5% solution from domesticated Bombyx mori silkworm (average M_w_ = 100 kDa) were purchased from Sigma-Aldrich, Darmstadt, Germany. The chemical structures of Triton X-100 and farnesol are presented in Figure 2. 

3,10-bis(3,5-dimethoxyphenyl)perylene (BDMP, synthetic route shown in Figure S3a, SI) and meso-tetraphenyl-tetrabenzo porphyrin palladium(II) (PdTBP, synthetic route shown in Figure S3b, SI) were synthesized as part of a previous work of our group [36].

### 2.2. Micelle Preparation

TTA–UC micelles. First, 1 × 10^−3^ M BDMP and 1 × 10^−4^ M PdTBP stock solutions in toluene were prepared. An amount of 500 µL of the stock solution of BDMP was mixed together with 250 µL of the stock solution of PdTBP and the solvent was evaporated under vacuum to dryness. The residue was dissolved in THF, 10% wt. Triton X-100 (5 g) aqueous solution, and deionized water (5 g). The mixture was homogenized using IKA^®^ Vortex Genius 3, Staufen, Germany. THF was removed by rotary evaporation, lowering the pressure from 200 mbar to 70 mbar and T = 40 °C. After the evaporation step, no material precipitation was observed. 

SSOS micelles. Farnesol (50 mg), 10% wt. Triton X-100 (5 g), and deionized water (5 g) were homogenized using a Hettich Heating Thermoshaker MHR23, Bäch, Switzerland (300 rpm, 37 °C, overnight). 

After an optimization procedure, namely testing different volume ratios of the two types of micelles, the highest temperature sensitivity was obtained for the volume ratio of 6 vol. parts (TTA–UC micelles)/5 vol. parts (SSOS micelles). All further experiments were performed with this sample composition. 

The hydrodynamic size of the micelles was determined by dynamic light scattering (DLS). The measurements were performed on an ALV spectrometer consisting of a goniometer and an ALV-5004 multiple tau full-digital correlator (320 channels, Langen/Hessen, Germany), which allowed measurements over an angular range from 30° to 150°. The temperature controlled light-scattering measurements were performed on the unfiltered samples at an observation angle of 90° at temperatures ranging from 15 °C to 42 °C (obtained by a thermostat, Julabo GmbH, Seelbach, Germany). The excitation wavelength of the DLS device was λ = 632.8 nm (excitation source, a HeNe laser), which overlapped significantly with the Q-band absorption of the sensitizer. Therefore, “TTA-UC” micelles, containing no sensitizer, were prepared in order to be measured by the DLS technique, keeping the amount of emitter materials (BDMP) unchanged. Since the concentration of the sensitizer was relatively low (1 × 10^−4^ M), the temperature dependence of the TTA-UC micelles was predetermined by the amount of the surfactant and the emitter. The temperature dependence of the hydrodynamic radius for the studied micelles is shown in Figure S2, SI. 

### 2.3. Hydrogel Preparation

The silk fibroin solution, 5% wt., was used as supplied, without any further purification. The 1% wt. agarose stock solution was prepared. Then, 600 µL of TTA–UC micelles and 500 µL of SSOS micelles were mixed. Then, the 200 µL silk fibroin solution and the 500 µL agarose stock solution were added to the micellar mixture at ≈50 °C and pipetted to obtain a homogeneous mixture. The mixture was immediately gently spin-coated onto a FluoroDish^®^, World Precision Instruments, Friedberg, Germany cell culture dish with an optical grade glass bottom. The hydrogels were kept at room temperature for 2 h before measurement.

### 2.4. Spectroscopic Measurements

**Figure 3:**
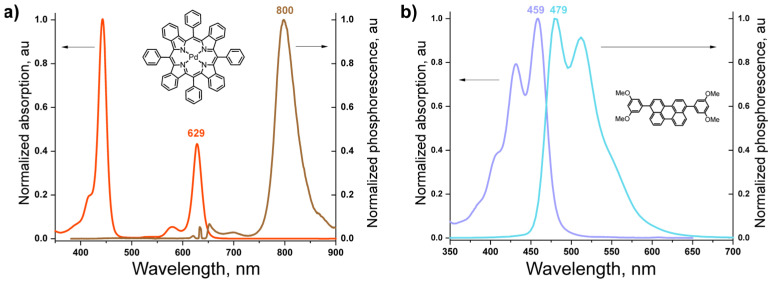
(**a**) Normalized absorption spectrum (red line) and normalized luminescence spectrum (brown line, excitation wavelength λ = 635 nm) of the sensitizer molecule in toluene solution; (**b**) normalized absorption spectrum (violet line) and normalized fluorescence spectrum (blue line, excitation wavelength λ = 459 nm) of the emitter molecule in toluene solution. Insets: structure of meso-tetraphenyl-tetrabenzo porphyrin palladium(II) (PdTBP) sensitizer (**a**), structure of 3,10-bis(3,5-dimethoxyphenyl)perylene (BDMP) emitter (**b**).

A Duetta™, HORIBA Inc., Darmstadt, Germany spectrometer was used to measure the absorption and fluorescence spectra of BDMP and PdTBP (Figure 3). The temperature dependence of the TTA–UC signals was recorded using a home-build setup (Figure S4, SI), as described previously [25]. 

Scanning electron microscopy (SEM) studies were performed by a field emission microscope (LEO (Zeiss) 1530 Gemini, Oberkochen, Germany), working at an accelerating voltage of 170 V. The silica wafers were cleaned in a plasma oven prior to use.

## 3. Results and Discussion

### 3.1. Micellar Systems

**Figure 4:**
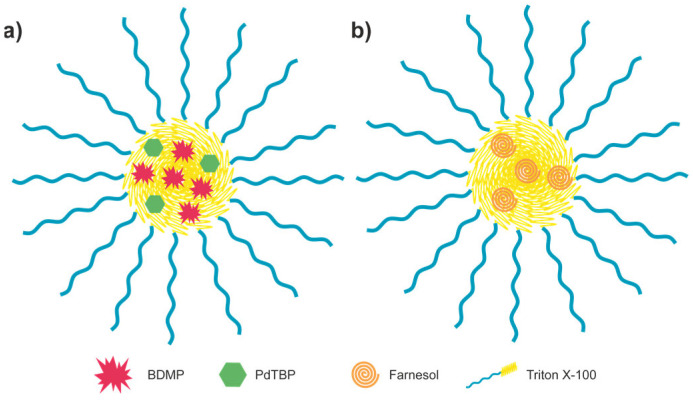
Scheme of the micellar system developed in this work: (**a**) TTA–UC micelle, (**b**) SSOS micelle.

Typically, the sensitizer and the emitter molecules are loaded into the same micellar system [32,37]. Here, the idea is to separate the sensing function (via TTA–UC) and the singlet oxygen protection function (via the formation of peroxide of acyclic terpene alcohol, farnesol, Figure 3b) in order to optimize both processes independently and to ensure unprecedented temperature sensitivity. The first group of micelles will incorporate the TTA–UC active materials, and the second, the sacrificial singlet oxygen scavenger (Figure 4).

### 3.2. TTA–UC in Hydrogel

Hydrogels are known to have most applications in the biomedical field, depending on their properties such as radiation sensitivity [38], biochemistry/bioengineering [39], drug delivery, wound healing [40], etc. 

The materials used for their composition are mainly polymers. A natural polymer representative is the silk of the silkworm Bombyx mori, which consists of two protein components: fibroin and sericin. The former is the structural protein of silk fibers and the second one is the water-soluble glue that binds the fibroin fibers together [35]. Silk scaffolds have been successfully used in wound healing and in tissue engineering [41]. 

There is also a variety of methods used for creating agarose hydrogels. In addition, there are several approaches to create a hydrogel from an aqueous solution of silkworm silk fibroin, including vortexing or sonication of silk fibroin [42], electrogelation [43], crosslinking silk fibroin with genipin [44], or physically crosslinking agarose hydrogels [45]. However, most of them use either toxic organic solvents or high temperature and/or high mechanical stress, which makes them unsuitable for the UC micellar system. 

**Figure 5:**
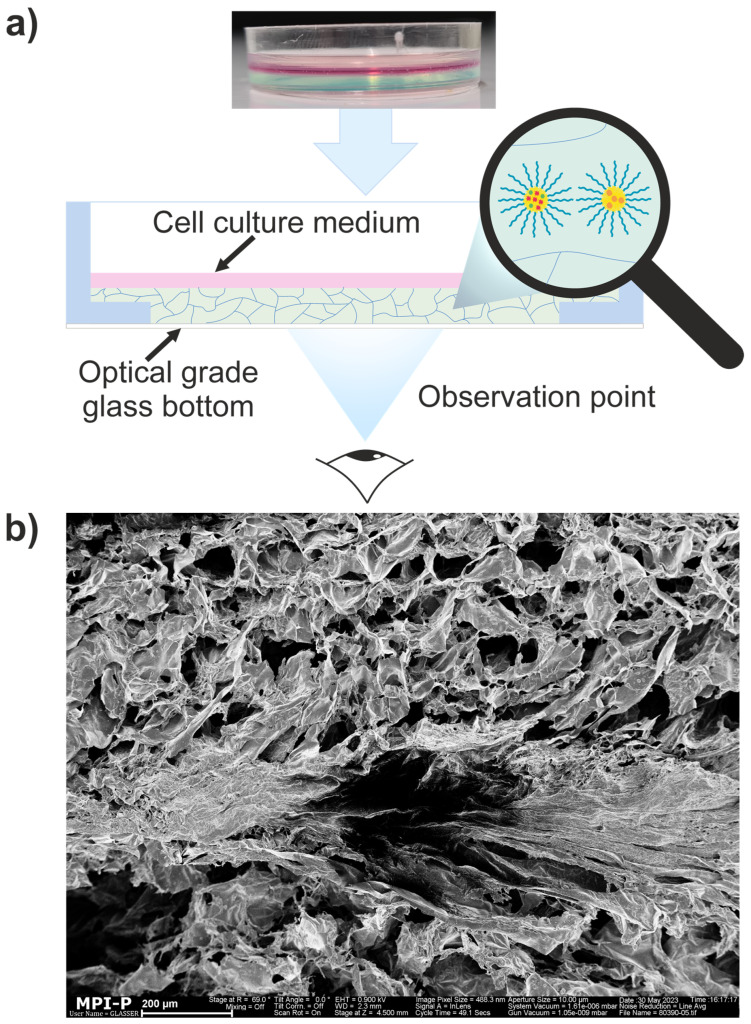
(**a**) Cartoon illustrating the observation setup, (**b**) SEM image of freeze-dried agarose/silk fibroin hydrogel showing its structure. The scale bar is 200 μm.

Agarose/silk fibroin hydrogels were prepared under mild temperature (20–50 °C) and gentle mechanical conditions (low speed stirring, moderate spin-coating rate, less than 100 rpm). A FluoroDish^®^ cell culture dish with an optical grade glass bottom (125 µm thick) for shorter working distances, larger numerical aperture, and higher magnification was used as the mechanical container of the sample (Figure 5a). This allowed for the straightforward further application of immersion objectives (HC PL APO 63×/1.40 OIL CS2, Leica Microsystems, Wetzlar, Germany); also, a tabletop incubator (P-Set 2000, PECON GmbH, Erbach, Germany), where different environmental conditions such as hypoxia up to normoxia can be simulated. Additionally, the optical quality flat bottom of the FluoroDish^®^ optimizes the heat transfer and is widely used for growing cell cultures. As is shown on the SEM image (Figure 5b), the pores of the hydrogel were approximately 20–80 µm. 

### 3.3. Creating a Calibration Curve

All experiments were performed in an oxygen-saturated environment, which inevitably determined the high concentration of molecular oxygen actively diffusing through the hydrogel surface. It is known that oxygen diffusion increases significantly with increasing the temperature [46]. In the case of non-ionic surfactant micelles, the oxygen permeability of the UC micelles is predetermined and cannot be optimized independently. Therefore, in order to achieve a relatively stationary intensity of the dF and the rPh signals, which is necessary to obtain an unambiguous and reproducible temperature calibration curve, a careful optimization of the triplet state generation rate is required. The triplet state generation is directly related to the excitation intensity. At the optimal excitation intensity (for the given sample composition), the oxygen permeation rate is much lower than the chemical binding rate of singlet oxygen over the optically evaluated spot. Therefore, after a short initial period, the entire oxygen content is consumed. Thus, during the further excitation, the optically assessed spot is practically oxygen free and the temperature-sensing procedure can be performed in a sustained and reproducible manner.

**Figure 6:**
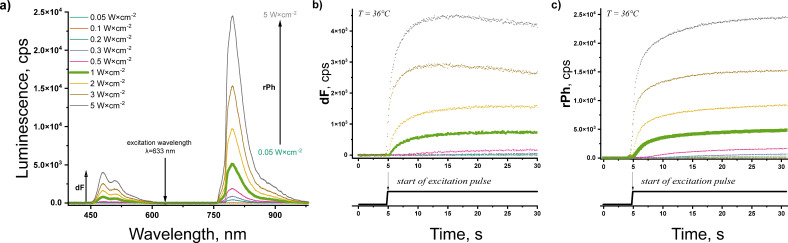
(**a**) Dependence of the luminescence spectra of the studied micellar system embedded in a spin-coated agarose/silk fibroin hydrogel on the excitation intensity, obtained 15 s after excitation start. (**b**) Time-resolved dependence of the signal of dF (λ = 479 nm) on excitation intensity. (**c**) Time-resolved dependence of the signal of rPh (λ = 800 nm) on excitation intensity. Conditions: constant sample temperature T = 36 °C; ambient environment (O_2_ > 20% vol.); excitation wavelength λ = 632,8 nm; excitation source HeNe laser; cw, excitation; the excitation laser is rejected by notch filter designed for λ = 633 nm; water layer on the top of the hydrogel, 1200 µm; sample composition: spin-coated agarose/silk fibroin hydrogel, 33% vol. TTA–UC micelles (PdTBP/BDMP)/28% vol. SOSS micelles (farnesol)/11% vol. silk fibroin/28% vol. agarose.

Figure 6a shows the dependence of the luminescence of the sample on the excitation intensity, along with the time-resolved signals of dF (Figure 6b) and rPh (Figure 6c) for a constant sample temperature of 36 °C. It is evident that, at a certain moment (almost 15 s after the start of excitation) and at an intensity of 1 W·cm^−2^, both signals—dF and rPh—reach stationary values. However, for the given oxygen contamination, for excitation intensities higher than this value, both optical signals undergo a decrease; on the other hand, for excitation intensities lower than 1 W·cm^−2^ and low sample temperatures (lower than 25 °C, not shown here), the dF signal is relatively low. Simultaneously, for intensities higher than 1 W·cm^−2^, instabilities of the optical signals (for the specific sample composition) are observed; the signal of rPh demonstrates a slow increase, whereas the signal of dF shows a substantial decrease. Therefore, in the further experiment, the excitation intensity is kept constant at 1 W·cm^−2^, Figure 6b (the green line) and Figure 6c (the green line). The quantum yield (QY) of the used TTA-UC micellar system (steady-state values, oxygen-free environment, i.e., nitrogen-filled glove box, less than 2 ppm O_2_, classical definition [25]) is 0.024.

**Figure 7:**
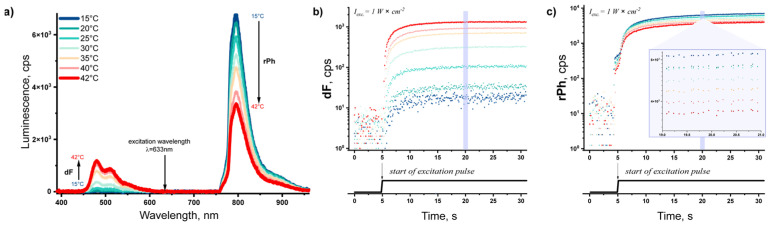
(**a**) Dependence of the luminescence spectra of the studied micellar system embedded in a spin-coated agarose/silk fibroin hydrogel on the sample temperature; (**b**) time-resolved dependence of the signal of dF (λ = 479 nm) on the sample temperature; (**c**) time-resolved dependence of the signal of rPh (λ = 800 nm) on the sample temperature. Conditions: constant excitation intensity 1 W·cm^−2^; ambient environment (O_2_ > 20% vol.); excitation wavelength λ = 632.8 nm; excitation source a HeNe laser; cw, excitation; the excitation laser is rejected by notch filter designed for λ = 633 nm; water layer on the top of the hydrogel, 1200 µm; sample composition: spin-coated agarose/silk fibroin hydrogel, 33% vol. TTA–UC micelles (PdTBP/BDMP)/28% vol. SOSS micelles (farnesol)/11% vol. silk fibroin/28% vol. agarose.

Figure 7a shows the dependence of the luminescence of the sample on the sample temperature, and the time-resolved signals of dF (Figure 7b) and rPh (Figure 7c) for a constant excitation intensity of 1 W·cm^−2^. As evident from Figure 7b, the signal of dF increases monotonically with the increasing sample temperature, while the signal of rPh (Figure 7c) decreases monotonically with the increasing sample temperature. 

**Figure 8:**
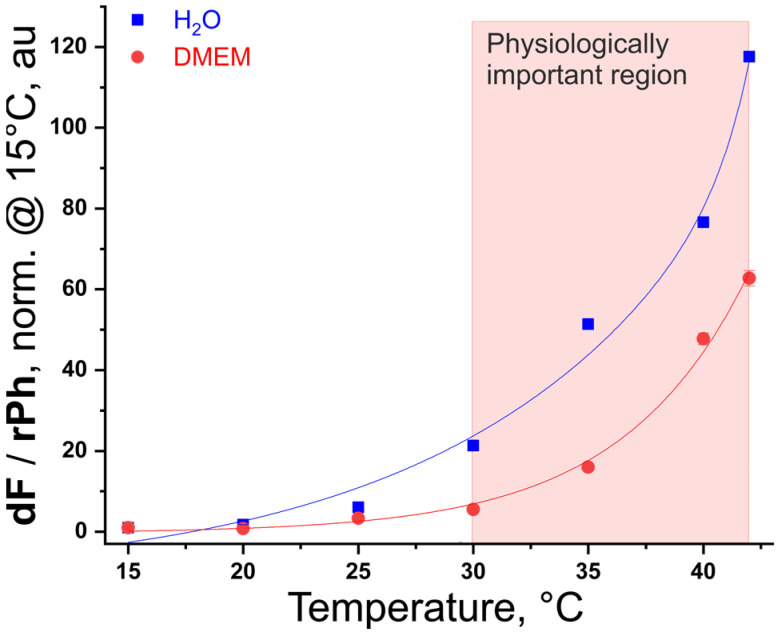
Temperature calibration curve: dependence of the ratio dF/rPh for the studied TTA–UC micellar system embedded in an agarose/silk fibroin hydrogel on the sample temperature. The ratio is normalized at 15 °C. Conditions and sample composition are the same as in Figure 7 (exception, the cover layer for blue line is 1200 µm water; the cover layer for red line is 1200 µm DMEM). Red and blue curves are guides for the eye. Sample composition is the same as in Figure 7.

This unique experimental result allows to create a ratiometric temperature calibration curve (Figure 8, the blue line), which demonstrates more than 100 times the change of the ratio dF/rPh for a temperature interval of ∆T = 15–42 °C when the liquid layer on the top of the agarose/silk fibroin translucent hydrogel is pure water. The ratio dF/rPh is calculated using the momentum values for each signal, obtained at t = 15 s after the start of the excitation. Normalization at 15 °C means that the ratio dF/rPh at temperature T = 15 °C is set to 1. This rule applies to all calibration curves reported in this work.

### 3.4. TTA-UC in Dulbecco’s Modified Eagle’s Medium

In order to verify the potential application of this TTA–UC sensing system for biological samples, we demonstrate an efficient annihilation upconversion in a hydrogel, completely covered with cell culture medium, which is commonly used in cell culture procedures. For our experiments, the cell culture medium consists of 90% DMEM (Dulbecco’s modified Eagle’s medium) high glucose [Gibco] (glutamine inside) + 10% h.i. FBS, +1% pen/strep. For simplicity, we will call this composition DMEM (a photograph of the DMEM-covered sample is shown in Figure 5a, the top image). The used DMEM contains a pH-sensitive dye (phenol red). It is important to note that the absorption spectrum of that compound demonstrates two isosbestic points at λ = 365 nm and λ = 477 nm [47], with substantially low absorption. The emission maximum of the signal of the dF (λ = 479 nm) overlaps almost perfectly with the red-shifted isosbestic point of the phenol red, guaranteeing minimal optical losses caused by re-absorption. Since neither the excitation wavelength λ = 632.8 nm (a HeNe laser) nor the emission maximum of the rPh signal (λ = 800 nm) overlap with the absorption spectrum of phenol red, no further re-absorption complications can be expected. Consequently, the enrichment of the cell culture procedure with the possibility to sense the temperature by the process of TTA-UC does not lead to unwanted experimental complications or loss of standard features. 

Correspondingly, the dependence of the hydrogel luminescence on the sample temperature, along with the temperature dependence of the time-resolved signals of dF and rPh for a constant excitation intensity of 1 W·cm^−2^ and sensing hydrogel covered with a 1200 µm layer of DMEM, are shown in SI Figure S1. The temperature calibration curve (Figure 8, the red line), derived from the data presented in Figure S1, demonstrates a change more than 60 times of the ratio dF/rPh for a temperature interval of ∆T = 15–42 °C. Here, we demonstrate for the first time the sensing properties of a DMEM-covered TTA–UC translucent hydrogel. The pink-colored rectangle in Figure 1 represents the physiologically important temperature window of (∆T_physio_ = 30–42 °C), centered around the temperature T~36 °C. Exactly for this temperature region, the ratio dF/rPh is changed more than 10 times (dF/rPh = 5.47 at T = 30 °C, consequently dF/rPh = 62.7 at T = 42 °C). Taking into account the low optical noise, at the level of ±2% demonstrated experimentally, the archived temperature sensitivity is better than 0.5 K.

Triton X-100 is a surfactant with a relatively low molecular weight, HLB = 13,4, and is well soluble in water. When the water layer on top of the sample is exchanged with a surfactant-free solution (1200 µm thick, required for cell culture breeding), we observe a slow aging of the sample; the emissive properties of the TTA-UC system has decreased. For instance, 72 h after the gel preparation (the water layer on top of the gel sample is exchanged three times, i.e., every 24 h) the TTA-UC emission drops e times (2.73 times).

**Figure 9:**
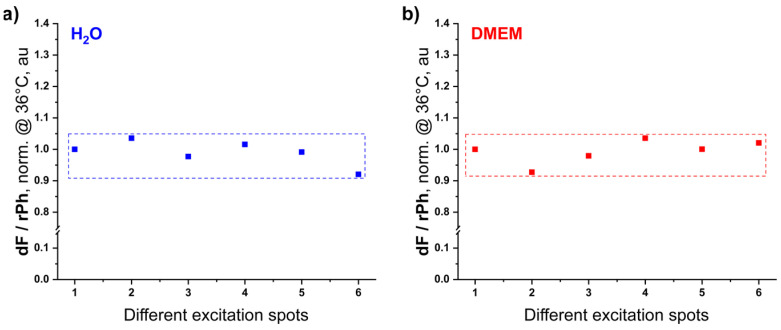
Comparison between temperature dependence of the ratio dF/rPh (normalized at 36 °C) for different excitation spots in the studied micellar system embedded in an agarose/silk fibroin hydrogel in the presence of (**a**) H_2_O and (**b**) DMEM. The excitation spots are at a distance of 3500 µm in both horizontal directions. Conditions and sample composition are the same as in Figure 7; sample composition is the same as in Figure 7.

The remarkable reproducibility of the temperature calibration curve is demonstrated in Figure 9. The ratio dF/rPh is measured for different excitation spots, keeping the sample temperature (36 °C) and the excitation intensity (1 W·cm^−2^) constant. The ratio dF/rPh also varies for water-covered hydrogel, as for DMEM-covered hydrogel, less than ±4%. The variations of the ratio dF/rPh can be explained by the peculiarity of the oxygen diffusion, and by the minimal thickness variations of the hydrogel.

## 4. Conclusions

Agarose/silk fibroin translucent hydrogels embedding non-ionic micellar systems containing energetically optimized annihilation couples potentially serving as a T-sensing scaffold independently of the local O_2_ concentration were created. The long-term and reproducible operation of the TTA–UC molecular system in an oxygen-saturated environment (O_2_ > 20% vol., above the water surface of the FluoroDish^®^) were verified. Temperature calibration curves, measured in different physical points of the agarose/silk fibroin hydrogel, having remarkable reproducibility (better than ±4%) were shown. For the first time, the temperature-sensing potential of the TTA–UC translucent hydrogel covered by Dulbecco’s modified Eagle’s medium was demonstrated. For the physiologically important region (∆T_physio_ = 30–42 °C) centered around the temperature T~36 °C, the ratio dF/rPh of the DMEM-containing sample changed more than 10 times and a sensitivity better than 0.5 K was obtained. This opens up the prospect of using the agarose/silk fibroin hydrogel as a minimally invasive temperature-sensing tool. Furthermore, the ability to tune the mechanical properties of the agarose/silk fibroin hydrogel allows it to serve as a scaffold for different cell types.

## Data Availability

Data available upon reasonable request.

## Supplementary Materials

The following supporting information can be downloaded at https://www.mdpi.com/article/10.3390/nano15020123/s1: Figure S1: (a) Dependence of the luminescence spectra of the studied micellar system embedded in a spin-coated agarose/silk fibroin hydrogel on the sample temperature; (b) time-resolved dependence of the signal of dF (λ = 479 nm) on the sample temperature; (c) time-resolved dependence of the signal of rPh (λ = 800 nm) on the sample temperature. Conditions: constant excitation intensity 1 W·cm^−2^; ambient environment (O_2_ > 20% vol.); excitation wavelength λ = 632,8 nm; cw, excitation; the excitation laser is rejected by notch filter designed for λ = 633 nm; DMEM layer on the top of the hydrogel, 1200 µm; sample composition: spin-coated agarose/silk fibroin hydrogel, 33% vol. TTA–UC micelles (PdTBP/BDMP)/28% vol. SOSS micelles (farnesol)/11% vol. silk fibroin/28% vol. agarose. Figure S2: Temperature dependence of the hydrodynamic radius for the studied micelles: (a) “TTA–UC” micelles and (b) SSOS micelles. Remarks: Measurements were performed for scattering angle 90°; (c) schematic representation of the proposed mechanism. Remarks: Red curve is the guide for the eye for the temperature calibration curve; dependence of the ratio dF/rPh for the studied TTA–UC micellar system embedded in an agarose/silk fibroin hydrogel on the sample temperature. (Figure 8) The ratio is normalized at 15 °C. Conditions and sample composition are the same as in Figure 7, the cover layer for red line is 1200 µm DMEM; Figure S3a: Synthesis schema of 3,10-bis(3,5-dimethoxyphenyl)perylene (BDMP); Figure S3b: Synthesis of PdTBP; Figure S4: Set-up for registration of the dynamical parameters of the TTA-UC in engineered atmosphere with actively controlled oxygen content and sample temperature.
