# Supplementary material for: Temperature Sensing in Agarose/Silk Fibroin Translucent Hydrogels: Preparation of an Environment for Long-Term Observation
Source: Nanomaterials (Basel). 2025 Jan 16;15(2):123. doi: 10.3390/nano15020123 (PMC11767501; doi:10.3390/nano15020123)
Supplement: Supplementary file 1 [file nanomaterials-15-00123-s001.zip › nanomaterials-3405215-suplementary.pdf]

Suplementary Materials

Temperature sensing in agarose/silk fibroin translucent hydrogels: Preparation of an environment for a long-term observation

Maria Micheva [1], Stanislav Baluschev [1, 2, *] and Katharina Landfester [1]

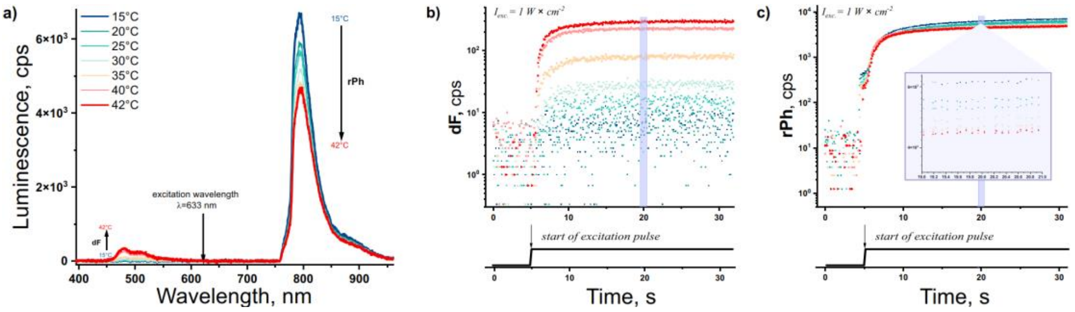

Figure S1: **(a)** Dependence of the luminescence spectra of the studied micellar system embedded in a spin coated agarose / silk fibroin hydrogel on the sample temperature; **(b)** Time-resolved dependence of the signal of dF (λ = 479 nm) on the sample temperature; **(c)** Time-resolved dependence of the signal of rPh (λ = 800 nm) on the sample temperature. Conditions: Constant excitation intensity 1 W×cm^{-2}; Ambient environment (O_2 > 20% vol.); Excitation wavelength λ = 632,8 nm; excitation source – HeNe laser; cw – excitation; The excitation laser is rejected by notch filter designed for λ = 633 nm**; DMEM layer** on the top of the hydrogel - 1200 μm; Sample composition: spin coated agarose / silk fibroin hydrogel: 33% vol. TTA–UC micelles (PdTBP / BDMP) / 28% vol. SOSS micelles (farnesol) / 11% vol. silk fibroin / 28% vol. agarose.

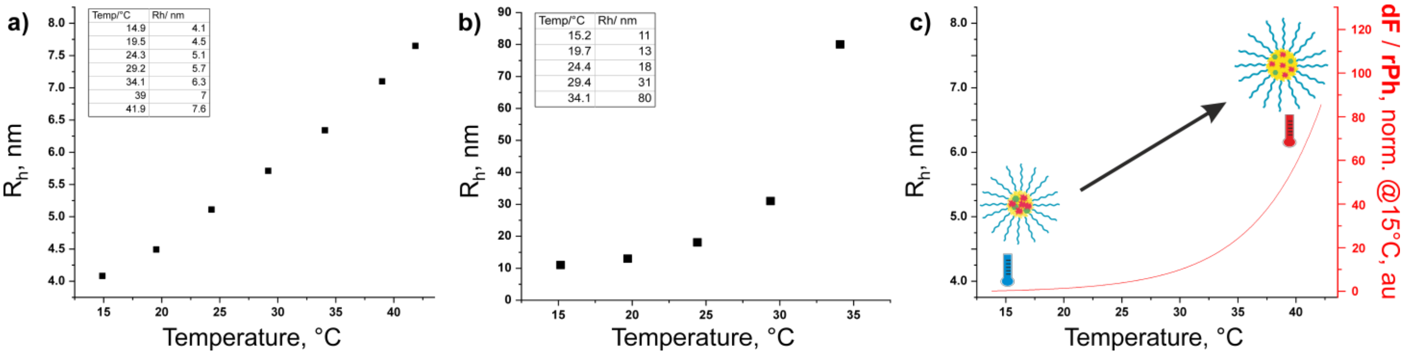

Figure S2: Temperature dependence of the hydrodynamic radius for the studied micelles: **(a)** “TTA–UC” micelles and **(b)** SSOS micelles. *Remarks*: Measurements were performed for scattering angle 90° **(c)** schematic representation of the proposed mechanism. *Remarks:* Red curve is the guide for the eye for the temperature calibration curve: dependence of the ratio dF / rPh for the studied TTA–UC micellar system embedded in an agarose / silk fibroin hydrogel on the sample temperature. (Figure 8) The ratio is normalized at 15 °C. Conditions and sample composition – the same as in Figure 7, the cover layer for red line is 1200 μm DMEM.

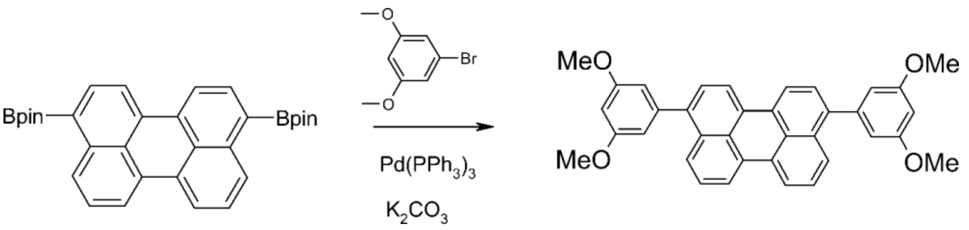

Figure S3a: Synthesis schema of 3,10-bis(3,5-dimethoxyphenyl)perylene (BDMP).

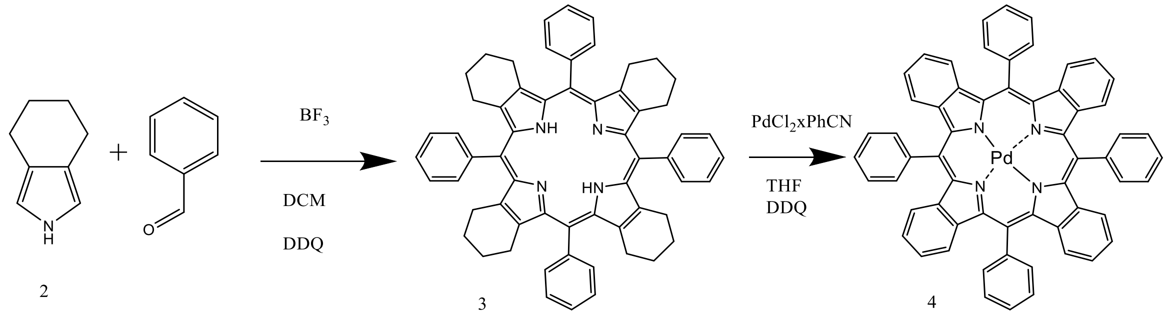

Figure S3b: Synthesis of PdTBP

Tetraphenyltetrabenzoporphyrin 3: Tetrahydroisoindole **2** (144 mg, 0.118 mmol, 1.0 equiv) was dissolved in dry DCM (100 ml). The reaction mixture was degassed for 20 min with argon. The stirred mixture was protected from light and stirred under argon. Benzaldehyde (125 mg, 0.118 mmol, 1.0 eq.) was added, and the reaction mixture was kept in the dark under argon and stirred for 10 min. BF_3 Et_2O (0.7 ml of 1% solution in CH_2Cl_2) was added in one portion, and the mixture was allowed to react at room temperature for 4 h. DDQ (110 mg, 41.8 mmol, 5.0 eq.) was added to the mixture in one portion, and the mixture was stirred at room temperature for 2 h. The resulting mixture was washed with 10% aqueous Na_2SO_3 (2x100 ml) and with water (2x100 ml).The combined organic phases were dried over Na_2SO_4. The solvent was evaporated, and the resulting residue was crystallized from methanol-DCM mixture. It was used in the next step without chromatographic purification.

PdTBP 4: An excess of $PdCl_2(PhCN)_2$ (140 mg, 0.36 mmol, 3.0 equiv) was added to a solution of **3** (100 mg, 0.12 mmol, 1.0 equiv) in THF (40 ml) with triethylamine (2 ml), and the mixture was refluxed for 2 h. DDQ (210 mg, 0.94 mmol, 10 equiv) was added, and the mixture was refluxed for 1 h. The solvent volume was reduced to 3 ml under vacuum. DCM (100 ml) was added, and the mixture was washed with aqueous 10% Na_2SO_3. The organic phase was dried over Na_2SO_4, and the solvent was evaporated. The crude product was purified by column chromatography on silica gel (eluent: toluene). Pure **4** was isolated as a dark-green solid with an overall yield of 70 mg (63%). ^{1}H-NMR ($CDCl_3$, 700 MHz) δ H = 8.27 (8H, dd, ^{3}J = 7.5, ^{4}J = 1.5), 7.92 (4H, tt, 3J = 7.5, 4J = 1.5), 7.85 (8H, dd, 3J = 7.5, 4J = 1.5), 7.21 (8H, dd, 3 J = 6.5, 4J = 3.0), 7.12 (8H, dd, 3J = 6.5, 4J = 3.0). ^{13}C-NMR ($CDCl_3$, 176 MHz) δ C = 142.4, 139.1, 138.5, 134.5, 129.7, 129.6, 125.8, 124.4, 118.6. MS (MALDI, dithranol matrix): m/z = 917.4 [M] + ; calculated for $C_{60}H_{36}N_4Pd$ 918.20.

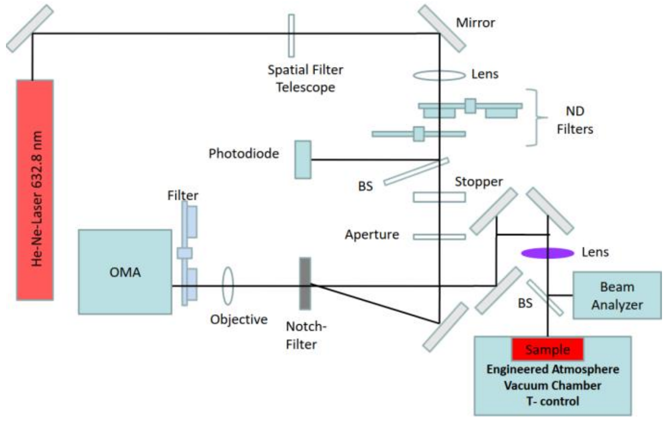

Figure S4: Set-up for registration of the dynamical parameters of the TTA-UC in engineered atmosphere with actively controlled oxygen content and sample temperature.

The laser beam pas through the spatial filter / telescope in order to reach TEM_{00} transversal intensity distribution with the desired defined beam diameter. A series of reflective neutral density (ND) filter (*Thorlabs Inc.*) placed on revolving optical holder (in order to keep the optical axes of the experiment unchanged) were used to attenuate smoothly the beam power. Afterward, the beam passed through the system of ultra-broadband mirrors (MaxMirror, Semrock Inc.) and finally focused by achromatic lens (NA = 0,24) onto the sample. A defocusing achromatic lens (the violet lens, Figure S4) placed on an electronically controlled μ-positioning stage (DL125 Delay Line Stage, *Newport Corp.*) controls the excitation spot diameter. The resulting excitation spot diameter is permanently controlled by a beam profiler (BP104-VIS, *Thorlabs Inc.*). The optical density of the all samples at the excitation wavelength is nearly 0.1; therefore the attenuation of the pump light can be neglected for the given thickness of the optical samples. The luminescence emission generated by the sample was collected with the same apochromatic lens, thus the excitation- and observation- spots are completely spatially overlapped. The emission of the excitation lasers was rejected by the appropriate notch filters: for instance by application of a notch filter designed for λ = 633 nm (FWHM ~ 29 nm, NF 03-633E-25, *Semrock Inc.*) rejection better than 106 times can be achieved. The *cw* - emission spectra were registered by Optical Multichannel Analyzer (*Hamamatsu Photonics Inc.*) or fiber – spectrometer (C10083CA, *Hamamatsu Photonics Inc.*) with absolute wavelength calibration and corrected spectral response. The sample chamber is equipped with input- and output optical windows, thus possible parasitic reflections for both, excitation and information optical signals caused by metal walls of the chamber, are completely avoided. The optical windows possess broad-band antireflection coating, keeping the unwonted reflections on levels less than 0,6% for all used wavelengths up to 1,1 μm. The sample is a subject of active temperature control performed by Peltier-element equipped with a hole with a diameter of 8×10^{-3} m. Thus, possible parasitic emissions / reflections caused by the ceramic surface of the Peltier-element are completely avoided. The Peltier-element current is actively controlled & stabilized by applying of a negative feedback electronic loop, thus any temperature at the interval of ΔT ~ 4 °C ÷ 54 °C with an accuracy of 0,1 °C can be realized and kept unchanged for enough long period (TCM Temperature Controller, *Electron Dynamics Ltd*). Stable operation of the Peltier-element temperature determination is achieved by appropriate remit of the parasitic heat created by means of recirculation chiller Ministat CC3, *Huber GmbH*.
